# Supplementary material for: Macro and microplastic pollution in Romania: addressing knowledge gaps and potential solutions under the circular economy framework
Source: PeerJ. 2024 Jun 24;12:e17546. doi: 10.7717/peerj.17546 (PMC11210483; doi:10.7717/peerj.17546)
Supplement: Supplemental Information 1 [file peerj-12-17546-s001.pdf]

Macro and microplastic pollution in Romania: Addressing knowledge gaps and potential solutions under the circular economy framework. PeerJ Life & Environment

Supplemental information_ Bibliometric analysis

Florin-Constantin MIHAI[1]*, Simona-Roxana ULMAN[2], Valeria POP[3,4],

[1]CERNESIM Environmental Research Center, Department of Exact Sciences and Natural Sciences, Institute of Interdisciplinary Research, "Alexandru Ioan Cuza" University of Iasi, Romania
[2] CERNESIM Environmental Research Center, Department of Exact Sciences and Natural Sciences, Institute of Interdisciplinary Research, "Alexandru Ioan Cuza" University of Iasi, Romania
[3] Doctoral School "Environmental Science", Babes-Bolyai University, Cluj-Napoca; Romania
[4]Research Institute for Sustainability and Disaster Management Based on High-Performance Computing, Faculty of Environmental Science and Engineering, Babeș-Bolyai University, Cluj-Napoca, Romania.

Corresponding Author:
Florin-Constantin MIHAI[1]
Carol I Blvd, No.11, 700506, Iaşi, Romania
Email address: mihai.florinconstantin@gmail.com;
https://orcid.org/0000-0002-1428-1021

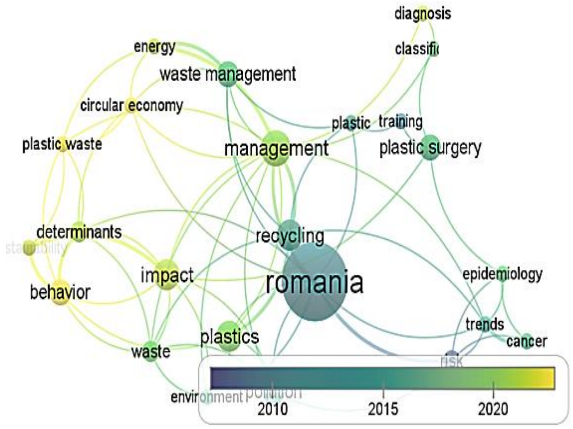

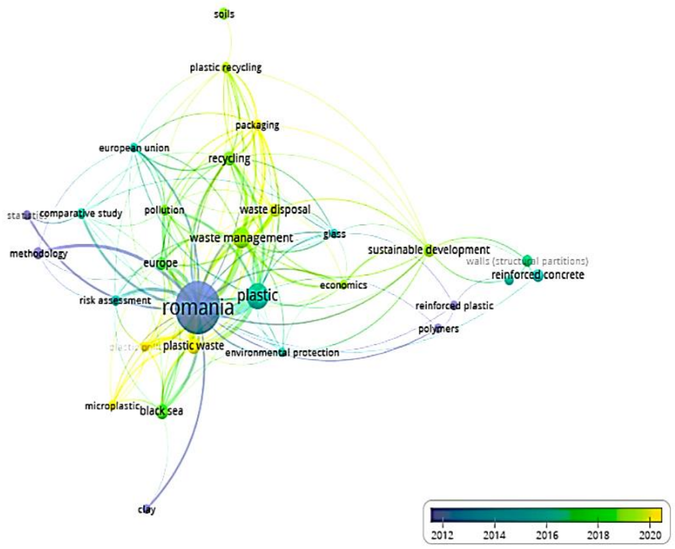

Figure 1 **(A) Overlay visualization - WoS** **(B) Overlay visualization – Scopus**

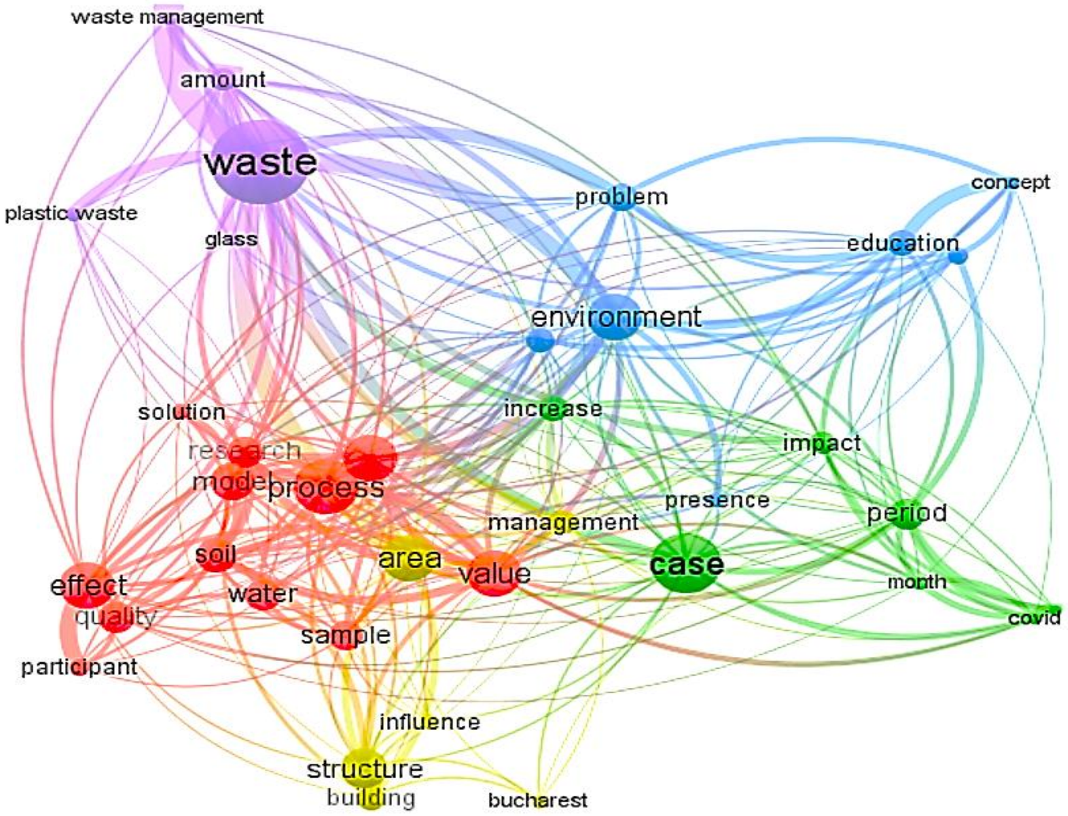

Figure 2 **(A) Network visualization – WoS**

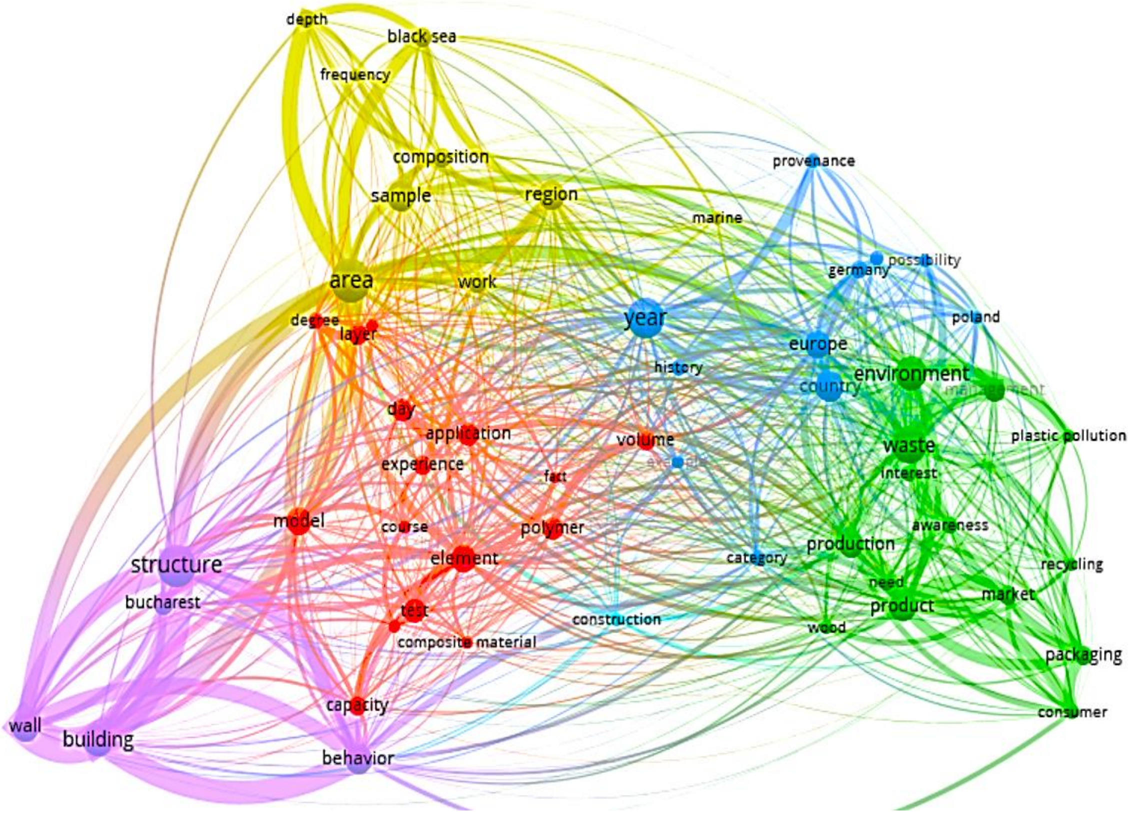

Figure 2 **(B) Network visualization – Scopus**

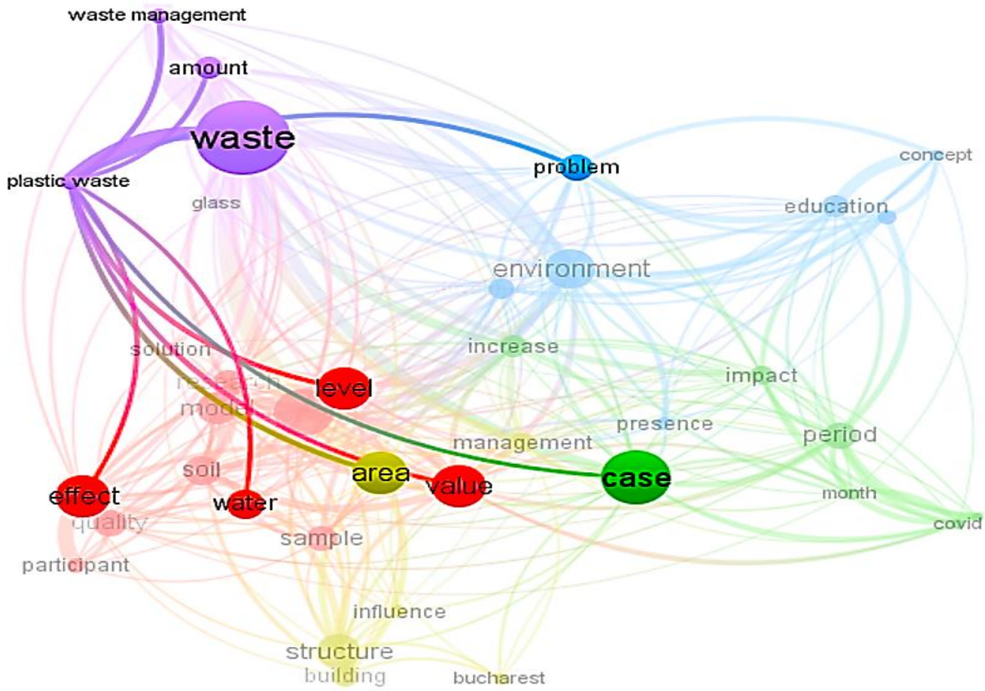

Figure 3 **(A) Network visualization – WoS with a focus on plastic waste**

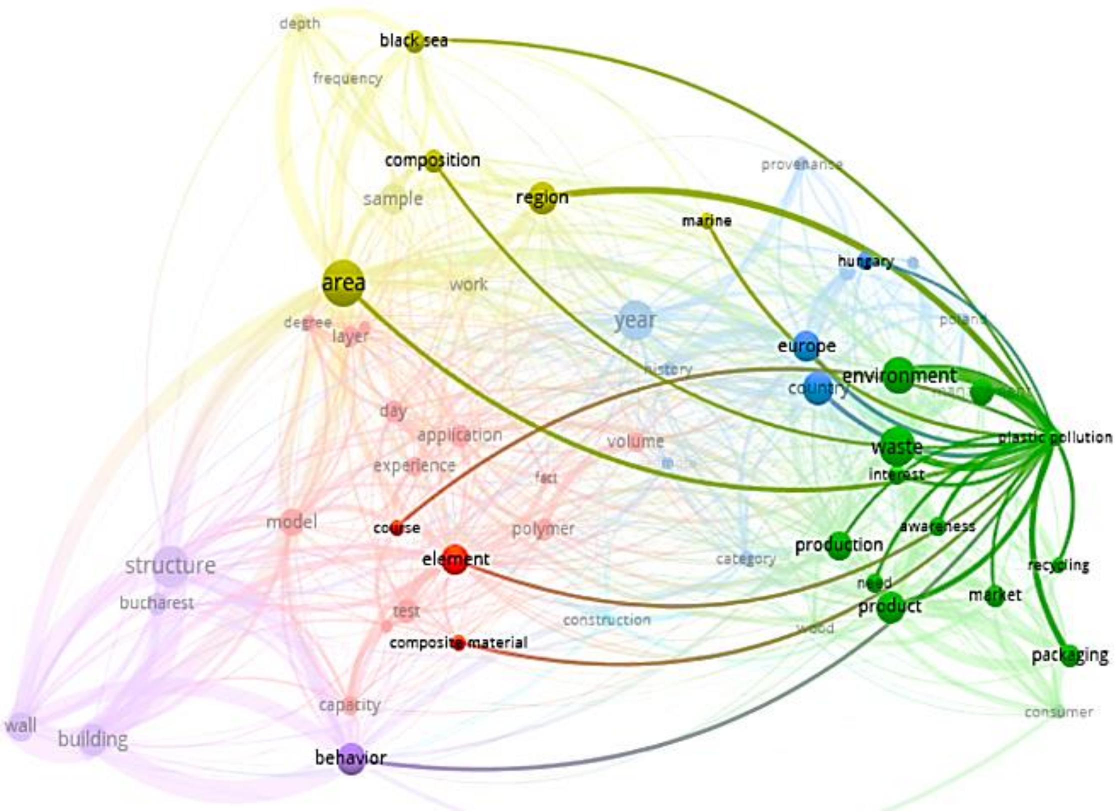

Figure 3 **(B) Network visualization – Scopus with a focus on plastic pollution**

The narrower search for the studies including plastic pollution and Romania terms

Web of Science: TS=Romania AND TS=Plastic pollution

15 papers published between 1997 and 2023 (1 paper in 1997; 1 paper in 2013; 1 paper in 2014; 1 paper in 2016; 3 papers in 2020; 3 papers in 2021; 2 papers in 2022; 3 papers in 2023 – from which 2 books, 1 conference paper, and 12 articles in journals)

Scopus: ”Romania” AND ”Plastic pollution”

27 papers published between 1997 and 2023 (1 paper in 1997; 1 paper in 2013; 2 paper in 2014; 3 papers in 2015; 2 papers in 2017; 2 papers in 2018; 2 papers in 2020; 4 papers in 2021; 6 papers in 2022; 4 papers in 2023 – from which 3 conference paper and 20 articles in journals)

Although only a small number of papers appropriate for our investigation was identified, we proceeded to apply a bibliometric analysis for trying to extract some conclusions from it. In this case of the narrower search for the studies including plastic pollution and Romania terms in the papers found in Web of Science, when generating the co-occurrence map based on keywords, four clusters were obtained as it might be observed from Fig. 4A: (i) plastics, Black Sea, marine litter; (ii) sea, pollution; (iii) pollutants, Romania; (iv) behavior, plastic pollution, waste, management. When emphasizing the period of publication, this search also reveals that the topic of our study is a new and under-investigated one, with papers especially concentrated

around the 2020 year (see Fig. 6A). The plastic pollution keyword co-occurred with the behaviour and waste keywords (Fig. 5A).

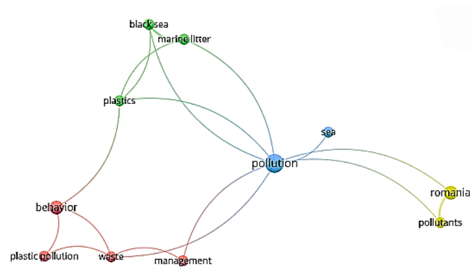

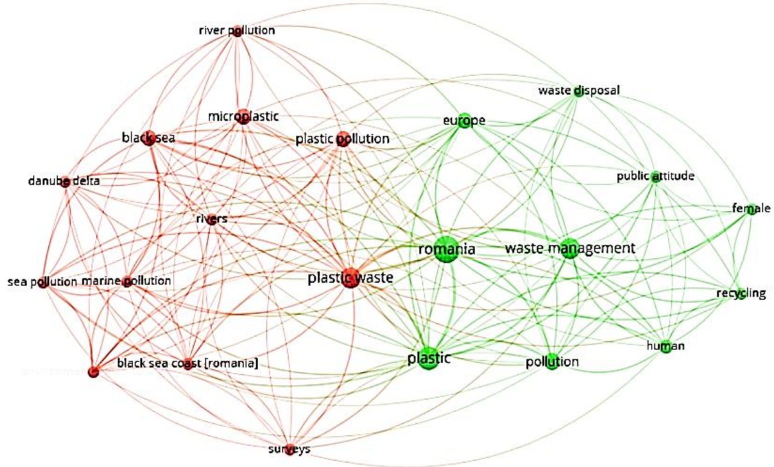

Figure 4 **(A) Network visualization – WoS**

(B) Network visualization – Scopus

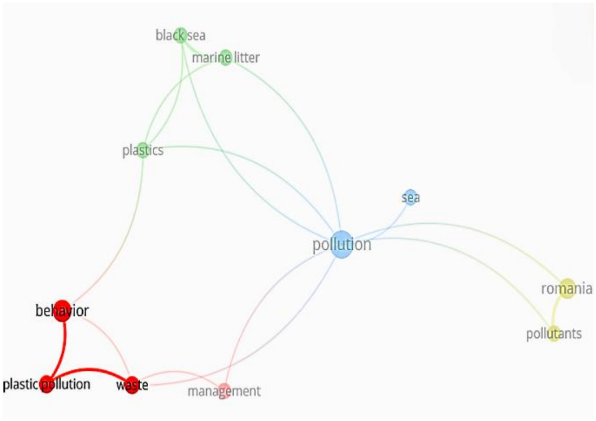

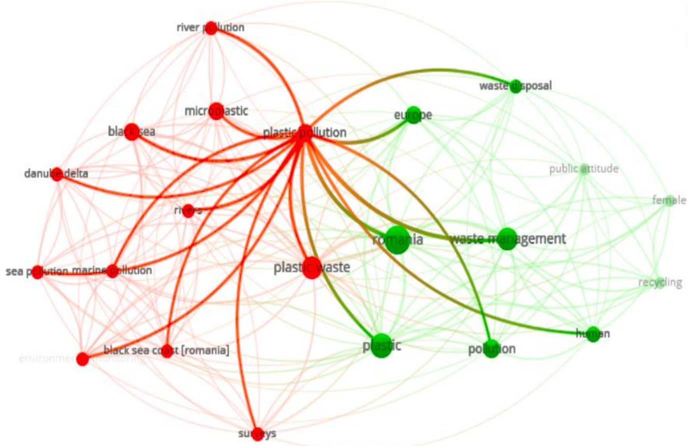

Figure 5 **(A) Network visualization with a focus on plastic pollution – WoS**

(B) Network visualization with a focus on plastic pollution – Scopus

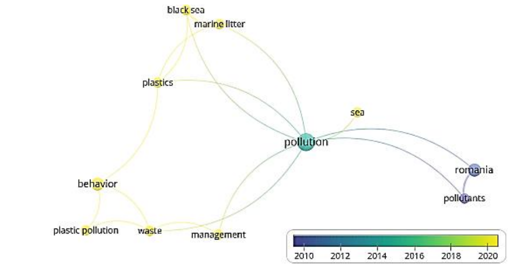

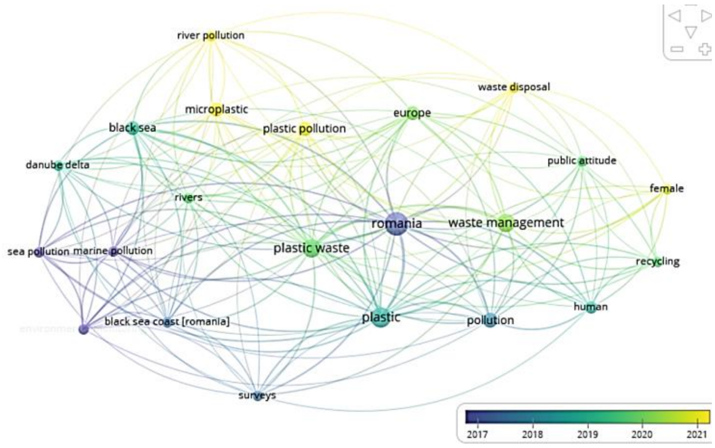

Figure 6 **(A) Overlay visualization - WoS**

(B) Overlay visualization - Scopus

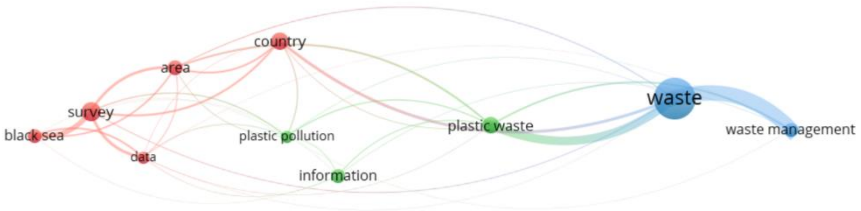

Figure 7 **(A) Network visualization – Wos**

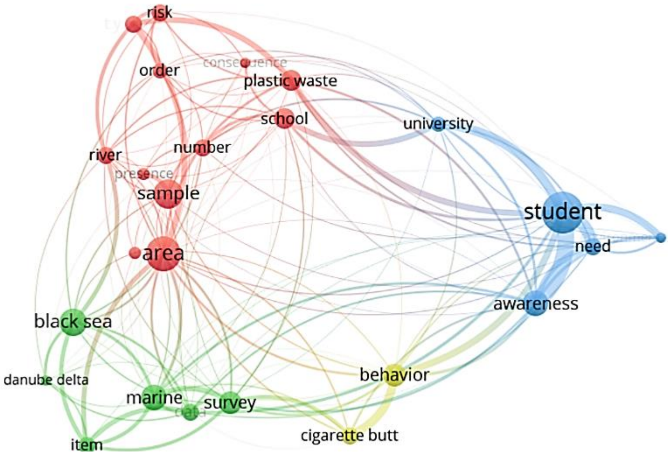

Figure 7 **(B) Network visualization – Scopus**

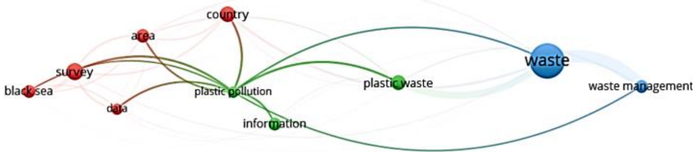

Figure 8 **(A) Network visualization with focus on plastic pollution – WoS**

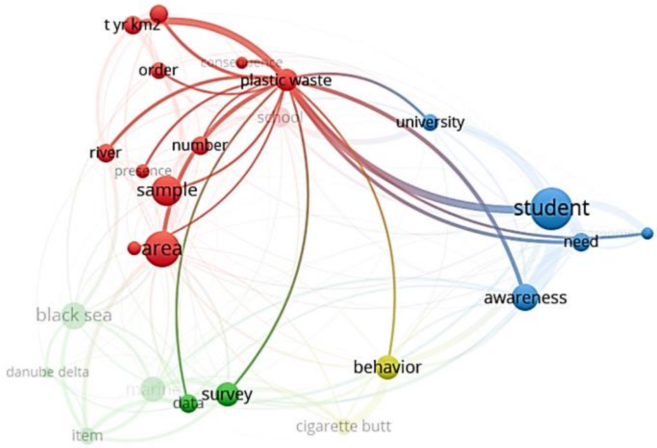

Figure 8 **(B) Network visualization with focus on plastic waste – Scopus**

Completing this analysis, the co-occurrences of the terms based on text from titles and abstracts of the studies included in the investigation from Web of Science were also investigated and

three clusters were formed as follows: (i) country, area, survey, data, Black Sea; (ii) plastic pollution, information, plastic waste; (iii) waste and waste management (see Fig. 7A).

In this case, the plastic pollution term is linked to terms such as data, Black Sea, survey, area, country, waste, plastic waste, or waste management (see Fig. 8A).

For the same narrower search, but applied to the studies found in Scopus (Fig. 4B), the co-occurrence map based on keywords contains two clusters as follows: (i) plastic waste, plastic pollution, microplastic, river pollution, Black Sea, marine pollution, Danube Delta, and (ii) Romania, waste management, plastic, Europe, waste disposal, recycling, public attitude, female. This search also reveals that the papers concentrated around our topic, with keywords related to plastic pollution were especially published around the 2020 year (see Fig. 6B). The plastic pollution keyword especially co-occurred with terms like plastic waste, surveys, Black Sea, marine/ sea pollution, Danube Delta, rivers, river pollution, waste disposal, Europe, waste management, human, or Romania (Fig. 5B). When analyzing the co-occurrences of the terms based on text from titles and abstracts of these studies, among the three clusters, the one containing items like plastic waste, area, risk, river, sample, and presence appears to be of interest for our research (see Figure 7B). In the case of this search, the plastic pollution term does not appear among the relevant terms from the titles and abstracts of the papers found in Scopus. Consequently, we directed our attention to plastic waste such as student, t yr km2, sample, area, need, awareness, university, survey, and river (see Fig. 8B).
