# Supplementary material for: Macro and microplastic pollution in Romania: addressing knowledge gaps and potential solutions under the circular economy framework
Source: PeerJ. 2024 Jun 24;12:e17546. doi: 10.7717/peerj.17546 (PMC11210483; doi:10.7717/peerj.17546)
Supplement: Supplemental Information 2 [file peerj-12-17546-s002.pdf]

Macro and microplastic pollution in Romania: Addressing knowledge gaps and potential solutions under the circular economy framework. PeerJ Life & Environment

Supplemental info _Table S1

Florin-Constantin MIHAI[1]*, Simona-Roxana ULMAN[2], Valeria POP[3,4],

[1]CERNESIM Environmental Research Center, Department of Exact Sciences and Natural Sciences, Institute of Interdisciplinary Research, "Alexandru Ioan Cuza" University of Iasi, Romania
[2] CERNESIM Environmental Research Center, Department of Exact Sciences and Natural Sciences, Institute of Interdisciplinary Research, "Alexandru Ioan Cuza" University of Iasi, Romania
[3] Doctoral School "Environmental Science", Babes-Bolyai University, Cluj-Napoca; Romania
[4]Research Institute for Sustainability and Disaster Management Based on High-Performance Computing, Faculty of Environmental Science and Engineering, Babeș-Bolyai University, Cluj-Napoca, Romania.

Corresponding Author:
Florin-Constantin MIHAI[1]
Carol I Blvd, No.11, 700506, Iaşi, Romania
Email address: mihai.florinconstantin@gmail.com;
https://orcid.org/0000-0002-1428-1021

Table S1. Policies to reduce MPs pollution leakage into the natural environment of Romania

Policy measure	Short details	Key reference
Prohibiting or restricting single-use plastics	Restricting or prohibiting the production, sale, and usage of single-use plastics such as bags, straws, and utensils	Directive (EU) 2019/904
Support for alternatives to plastic packaging materials	Encouraging the use of alternative materials or reusable products	Concordia (2022)
Implementation of DRS system	Improving capture rates of high-quality plastic packaging materials	Cliza and Spătaru-Negură (2021)
Source separation in five waste fractions	Improving capture rate of dry recyclables and biowaste to reduce waste volume and landfilling rates	Zero Waste Romania (2019)
EPR Policy	Implementing extended producer responsibility programs to hold them accountable for the entire life cycle of their products, including proper disposal	Ministry of Environment, Water and Forests (2022)
Water management and monitoring	(i) Investing in new more advanced wastewater treatment facilities to capture and filter MPs;<br>(ii) Identifying sources of Mps pollution as well as monitoring for a regular assessment of water quality	Talvitie et al. (2017)
Reserch and Innovation	(i) Supporting the research and development of innovative technologies to reduce Mps pollution, this field of research being still in the pioneering phase in Romania;<br>(ii) Providing financial support (packages and incentives for non-governmental organizations and research institutions) that work on solutions that support the reduction of MPs pollution;<br>(iii) Offering financial incentives and granting tax reductions for businesses that develop and adopt innovative solutions in Romania, to reduce Mps pollution	Act for Tomorrow (2021);<br>Mai Mult Verde Association (2023)
Public awareness and education	Launching public awareness campaigns (surveys) to educate citizens about the impact of MPs on the environment and the importance of responsible plastic use	Pop et al. (2023)
International collaboration	Romania collaborates with neighboring countries (Danube river basin) and Black Sea region countries to reduce transboundary plastic pollution and land-based plastic emissions to the marine environment	Kittner et al. (2022);<br>Liro et al., 2023
Legislation and enforcement	Strengthening existing legislation on plastic pollution and MPs and introducing new laws (transposed from the European level) specifically targeting MPs	European Commision (2023)
Zero waste business	Encouraging and supporting businesses and industries to adopt sustainable practices, such as reducing plastic packaging and incorporating circular economy principles	Puscaselu et al. (2021)

References

Act for Tomorrow (2021) Mapping Microplastic in Romanian Waters. https://actfortomorrow.ro/works/cartografierea-microplasticului-in-apele-romaniei/ (accessed 13 November 2023)

European Commission (2023) EU action against microplastics. https://environment.ec.europa.eu/system/files/2023-10/microplastics-brochure_final.pdf (accessed 10 November 2023)

Cliza MC, Spătaru-Negură LC (2021) Towards a Cleaner Planet-The Implementation of the Deposit Guarantee System in Romania. Perspectives of Law and Public Administration 10(1):54-64

Concordia (2022) Circular Economy in Romanian business. Toolkit for sustainable development. https://concordia.ro/noutati/documente-de-pozitie/economia-circulara-in-businessul-romanesc-mic-ghid-de-bune-practici-pentru-crestere-durabila (accessed 10 November 2023)

Directive (EU) 2019/904 of the European Parliament and of the Council of 5 June 2019 on the reduction of the impact of certain plastic products on the environment (Text with EEA relevance) https://eur-lex.europa.eu/eli/dir/2019/904/oj

Kittner M, Kerndorff A, Ricking M, Bednarz M, Obermaier N, Lukas M, Asenova M, Bordós G, Eisentraut P, Hohenblum P, Hudcova H, Humer F, István TG, Kirchner M, Marushevska O, Nemejcová D, Oswald P, Paunovic M, Sengl M, … Bannick CG (2022) Microplastics in the Danube River Basin: A First Comprehensive Screening with a Harmonized Analytical Approach. American Chemical Society (ACS), ACS ES&T Water 2(7):1174–1181 https://doi.org/10.1021/acsestwater.1c00439

Liro M, Zielonka A, van Emmerik TH, Grodzińska-Jurczak M, Liro J, Kiss T, Mihai FC (2023) Mountains of plastic: Mismanaged plastic waste along the Carpathian watercourses. Science of the Total Environment 888, 164058. https://doi.org/10.1016/j.scitotenv.2023.164058

Mai Mult Verde Association (2023) Study on the presence of plastic materials in the waters of the Danube, in Romania. https://cuapelecurate.ro/wp-content/uploads/2023/09/Studiu-microplastic_Asociatia-MaiMultVerde_2023.pdf (accessed 10 October 2023

Ministry of Environment 2022. Circular Economy Strategy. http://www.mmediu.ro/app/webroot/uploads/files/Strategia%20Nationala%20privind%20Economia%20Circulara%20-%20var%20finala.pdf (accessed 9 November 2023)

Puscaselu RG, Anchidin-Norocel L, Petraru A, Ursachi F (2021) Strategies and Challenges for Successful Implementation of Green Economy Concept: Edible Materials for Meat Products Packaging. Foods 10(12), 3035. https://doi.org/10.3390/foods10123035

Talvitie J, Mikola A, Koistinen A, Setälä O (2017) Solutions to microplastic pollution–Removal of microplastics from wastewater effluent with advanced wastewater treatment technologies. Water research 123:401-407. https://doi.org/10.1016/j.watres.2017.07.005

Pop V, Ozunu A, Petrescu DC, Stan AD, Petrescu-Mag RM (2023) The influence of media narratives on microplastics risk perception. PeerJ., 11, e16338. https://doi.org/10.7717/peerj.16338

Zero Waste Europe (2019) The Story of Salacea (Romania). https://zerowastecities.eu/bestpractice/the-story-of-salacea/ (accessed on 10 August 2023)
